# The European Board of Cardiovascular Perfusion statement on organ perfusion

**DOI:** 10.1093/icvts/ivaf007

**Published:** 2025-02-06

**Authors:** Gerdy Debeuckelaere, Adrian Bauer, Andreas Lemmers, Filip De Somer

**Affiliations:** Perfusion Department, Antwerp University Hospital, Edegem, Belgium; Perfusion Department, Evangelic Heartcenter, Coswig, Germany; Perfusion Department, Antwerp University Hospital, Edegem, Belgium; Perfusion Department, Ghent University Hospital, Ghent, Belgium

**Keywords:** organ perfusion, transplantation, European Board, perfusionist

Since 1991, the ‘European Board of Cardiovascular Perfusion (EBCP)’ has played a vital role in the European healthcare system by overseeing and promoting the field of cardiovascular clinical perfusion.

Through establishing standardized educational requirements and curricula, for perfusionists, the EBCP ensures high standards of patient care across Europe.

Over the consistent establishment of a ‘European Certificate in Cardiovascular Perfusion (ECCP)’, the EBCP ensures that perfusionists possess the necessary skills, knowledge and expertise to perform perfusions safely, effectively and based on scientific evidence.

‘ECCP perfusionists’ are specialized healthcare professionals responsible for operating different types of perfusion/gas exchange-related equipment, including heart-lung machine and other circulatory/respiratory assist devices and managing patients’ physiological status during cardiac and thoracic surgeries.

The ‘ECCP perfusionist’ works primarily within the framework of delegation from physicians, not substitution.

‘ECCP perfusionists’ are also involved in extracorporeal perfusions at the Intensive Care Unit and Heart Catheterization unit as well during resuscitations (eCPR) at the Emergency Department and transports of patients on extracorporeal support outside the hospital.

‘ECCP perfusionists’ have the leading expertise in the field of clinical perfusion in Europe and are therefore the predestined partners for the application of clinical perfusion procedures in Europe.

‘Organ perfusion’, also known as machine perfusion, is a clinical perfusion method used to preserve and improve the quality of donor organs for transplantation. Organ perfusion has been present in the European healthcare system since the early 2000s. This method extends the viability and increases the quality of donor organs inside or outside the body, improving outcomes for transplant recipients and addressing the shortage of suitable donor organs.

‘Organ perfusion’ includes different methods using a wide range of medical devices. Each of the various methods has its own characteristics. However, what remains universal is the use of a perfusion pump, a method of oxygenation and a series of tubes that together form an extracorporeal circuit.

Following the initial use of less complex procedures, more complex procedures have played an increasingly important role, particularly since the 2010s. In addition to hypothermic organ perfusion, normothermic modalities are showing promising results. The development of more highly complex modalities, in particular, shows that the possibilities of the procedure have not yet been reached. Compared to static cold storage, they offer a considerable advantage in terms of organ quality in Donation after Circulatory Death (DCD) and Donation after Brain Death (DBD).

‘Organ transplantation’ is a medical area in which different specialties work together to achieve a common goal: improving the quality of life of patients with end-stage organ failure.

As the only European organization that is involved in structured training (Perfusion program accreditation), continuous medical education (recertification process) and publication of (interdisciplinary) guidelines for clinical perfusion, the ‘European Board of Cardiovascular Perfusion’ wants to recommend that the use of any clinical perfusion machine in Europe should be in the expert hands of ECCP perfusionists, meaning that ECCP perfusionists are the preferred experts and a critical component of the multidisciplinary team. ECCP perfusionists should operate as well as take part in the decision-making of organ perfusion devices.

Just like in other fields, the addition of the expertise of ECCP perfusionists is of great value in the further development of machine/organ perfusion. This can take place in any form, i.e. in education and research but also in the clinical application of organ perfusion by ECCP perfusionists.

**Conflict of interest:** none declared.

## Data Availability

There are no new data associated with this article.

